# Sustained impact of UK FAST-test public education on response to stroke: a population-based time-series study

**DOI:** 10.1111/ijs.12484

**Published:** 2015-04-08

**Authors:** Frank J Wolters, Nicola L M Paul, Linxin Li, Peter M Rothwell

**Affiliations:** 1 Nuffield Department of Clinical Neurosciences, University of Oxford, John Radcliffe HospitalOxford, UK

**Keywords:** FAST, patient behavior, public education, stroke

## Abstract

**Background:**

Urgent assessment is essential after stroke. Several countries have had public education campaigns, based on the FAST (Face-Arm-Speech-Time) test to reduce delays in seeking attention. However, the impact of these campaigns on patient behavior is uncertain.

**Methods:**

We prospectively determined patient behavior after incident major stroke (NIHSS > 3) in a UK population based study (Oxford Vascular Study) before (2002–2008) and after (2009–2013) introduction of the FAST TV-campaign and assessed any sustained impact of campaign continuation.

**Results:**

Among 668 consecutive patients with major stroke, medical attention was sought by a bystander in 553 (89·6%). Patients were more likely to present directly to emergency services (OR = 2·18, 95%CI:1·54–3·09, *P* < 0·0001) after the campaign and to arrive at hospital within 3 h (OR = 2·18, 1·55–3·06, *P* < 0·0001). Median [IQR] time to seeking attention fell from 53 [15–265] to 31 [7–120] minutes (*P* = 0·005) and median time to hospital arrival from 185 [88–885] to 119 [78–256] minutes (*P* < 0·0001). On time-series analysis improvements in hospital arrival within 3 h and use of emergency medical services were significantly associated to initiation of the campaign (aOR = 3·11, 1·53–6·29, *P* = 0·002; and 2·22, 1·05–4·67, *P* = 0·036, respectively), independent of trend, age, sex, ethnicity, educational level, social class, prior stroke and stroke severity, and have been sustained to 2013.

**Conclusion:**

Delays to seeking and receiving medical attention after major stroke in the UK. fell strikingly in 2009, coinciding with the start of the FAST TV campaign. That medical attention was sought by a bystander in nearly 90% of cases illustrates the importance of mass-media public education rather than focused programs in high-risk groups for major stroke.

## Introduction

Fast medical assessment is essential after stroke, as the benefit of thrombolytic treatment declines rapidly over the first few hours following symptom onset 1,2. However, fast assessment is often hindered by pre-hospital delays in all countries from which data are available 3. The face-arm-speech-time (FAST) test was therefore devised as a tool to increase recognition of acute stroke by the public, patients and paramedics 4,5, and has since formed the basis of public education campaigns in many countries, including the UK, Ireland, USA, Australia and New Zealand, with variants in several non-English speaking countries.

The FAST test was used in an ongoing television public awareness campaign in the UK from 2009 onwards. Public awareness of the campaign was high 6, but previous studies of similar campaigns for stroke and other conditions have shown that increased public awareness seldom leads to changes in behavior 7–9. Three studies in the U.S. have used the FAST test as an education tool locally in small selected populations [383 women attending a beauty salon 10, 402 rural-dwelling adults 11, and 72 women in Massachusetts 12] and found that it increased recognition of stroke warning signs. Three retrospective studies reported routinely collected data with regard to national FAST campaigns, observing an increased number of ambulance dispatches for stroke since the FAST acronym was used in Australia 13, a non-sustained increase in emergency department attendance with stroke symptoms following FAST campaigns in Ireland 14, and an increase in the number of emergency department admissions for stroke and possibly thrombolysis rates in the UK 15. However, despite the large international investment in the FAST campaign, no published studies have yet assessed its impact on patient behavior at a population level, nor in the necessary detail to guide further public education campaigns.

We prospectively determined patient behavior after major stroke in an ongoing population based study (Oxford Vascular Study) from 2002 onwards. We now aimed to determine whether there has been any sustained impact of the FAST TV campaign on patient behavior at the population level in the UK, and whether impact was consistent for various factors influencing patient behavior, such as age, sex, education level and social class 3,16.

## Methods

The Oxford Vascular Study (OXVASC) is a population-based study of all acute vascular events, including stroke and TIA in about 92 000 individuals of all ages registered with 100 collaborating primary care physicians in nine general practices in the Oxfordshire, United Kingdom. The OXVASC study methods have been described previously 17. Briefly, multiple overlapping methods are used to achieve near complete ascertainment of all individuals with stroke or TIA (defined as symptom duration of less than 24 h). These include: (i) a daily, rapid access TIA and stroke clinic to which participating general practitioners (GPs) and the local emergency department refer individuals with suspected TIA or stroke whom they would not normally admit directly to hospital; (ii) daily searches of admissions to hospital wards and emergency department attendance; (iii) monthly searches of GP diagnostic coding and hospital discharge codes; (iv) monthly searches of all carotid imaging studies performed in local hospitals; (v) daily contact with hospital bereavement officers to identify patients brought into hospital dead or who died soon after arrival, and review of all death certificates in the study practices and ICD10 vascular death codes from the local Department of Public Health. This paper includes all consecutive consenting incident major stroke cases occurring out of hospital from April 1, 2002 up till March 31, 2013.

The vast majority of patients was admitted at the acute stroke service in the principal center serving the study population 17. Patients were consented (or assent was obtained from relatives) and seen by study physicians as soon as possible after initial presentation (usually the next working day) to determine their perception about the event and immediate response to symptoms, including dates and times of symptom onset (t_0_), when medical attention was sought and by whom, and the first contact with emergency medical services. Baseline characteristics, including demographic data and risk factors for stroke (such as hypertension and diabetes, based on prior diagnosis and current medication use), were recorded and assessments were made for severity of the event, using the National Institutes of Health Stroke Scale (NIHSS). Major stroke was defined as NIHSS > 3. Further data were acquired from medical records, ambulance sheets, GP referral letters and consultation notes.

If onset of stroke was during sleep, t_0_ was defined as time of awaking. In patients unable to call for help, t_0_ was considered as the moment another person noted their symptoms. Events were classified as ‘FAST positive’ when at least one symptom from the FAST-campaign (i.e. facial weakness, arm weakness or speech disturbance) was present at symptom onset. Strokes leading to loss of consciousness were not considered to be FAST-positive or -negative.

### FAST campaign in the UK

The main FAST public education television campaign in the UK ran from February–April 2009 with 8 weeks of national television advertisements. The ‘T’ in FAST was thereby re-designated ‘Time to call 999’ rather than ‘Test all 3’. The advertisement further depicted stroke as a fire, rapidly spreading in the head of an older adult. Repeated television campaigns ran intermittently for several weeks from October–December 2009, six weeks in March–April 2011 and four weeks in March 2012 and March 2013. The total campaign investment from 2009–2013 was 10·2 million pound sterling. The impact of the campaign as measured in the last two-years by television viewer ratings was 317 in 2013 and 274 in 2012 (1 television viewer rating equals 1% of the target audience of all adults >18 years of age; multiple views are counted cumulatively). In October 2005 there was a preceding small-scale 18-month public transport poster campaign.

### Analysis

Analyses included all (within the study period – i.e. patients with prior stroke before April 1, 2002 included) first major strokes occurring out of hospital during the study period, apart from analyses of patient perception, which excluded patients with reduced consciousness, event-related confusion or dysphasia. We analyzed patients' behavior before and after April 1, 2009 (i.e. six-years prior vs. four-years following the end of the first major television campaign) and stratified into study years.

Time from stroke onset to first seeking medical attention and the nature of the first medical attention sought (defined as ‘emergency’ – i.e. direct contact with ambulance services or presentation to an emergency department, vs. ‘non-emergency’ – i.e. first contact with a general practitioner or other local health care provider, such as ship- or airport medical services) were analyzed before vs. after April 1, 2009. Time from stroke onset to first seeking medical attention was compared as a covariate in a Kaplan–Meier analysis, by Mann–Whitney *U*-test, and by comparison of medians. We also compared the odds of seeking medical attention, and hospital arrival within the clinically relevant cut-off of 3 h by χ^2^-test and Mantel–Haenszel odds ratios. We furthermore identified impact on specific subgroups of the population, by separately assessing patients aged >75 years, men and women, those with higher educational levels [education beyond age 18 vs. further (16–18) and lower education (≤16)], and various social classes within the population, using the UK's 2007 indices of deprivation 18. Based on these indices, the electoral districts covering our population are less deprived than the rest of England, but still covering a broad range of deprivation, with 22% of our districts ranking in the lower third nationally. To further assess time trends, first contact with emergency vs. non-emergency services and hospital arrival within three-hours were analyzed for each year of the study, and subsequently adjusted for age, sex, ethnicity, prior stroke or TIA, NIHSS (classified 4–8; 9–15; 16–21; 21–42), educational level (as above), cohabiting, and social class (deprivation index) by means of segmented regression analysis 19, breaking down time to months and again using April 1, 2009 as change point. Missing values for covariates were imputed, using fivefold imputation (for details, please see Supporting information). Thrombolytic treatment rates were compared the four-years prior vs. four-years after April 1, 2009.

We classified perception of symptoms as correct (attributed to ‘stroke’, ‘TIA’, or ‘mini-stroke’) or incorrect (all else) and determined the proportion of patients with correct initial perception of symptoms before vs. after April 1, 2009 and the association between perception and time to seeking medical attention. We also determined the contribution of patient delay to total pre-hospital delay, by calculating for each patient the proportion of their total pre-hospital delay that was accounted for by time to first seeking medical attention. For patients presenting to emergency medical services, we also calculated the share of paramedic assessment time and ambulance transport time in the same way. Finally, sensitivity analysis were done comparing only the four-years immediately prior to April 1, 2009 vs. the subsequent four-years, assessing wake-up events separately, distinguishing left- and right-hemispheric events, and stroke severity. Analyses were done using IBM SPSS Statistics 21·0. Alpha (type 1 error) was set at 0·05.

## Results

Among 668 consecutive patients included in the study, 416 had a stroke pre-FAST and 252 post-FAST. Baseline characteristics are presented in Table 1. Data on the first medical healthcare provider contacted were obtained in 647 (96·9%) cases and the person who sought medical attention was known in 617 (92·4%) cases. We had no data on event time or time of seeking medical attention in 52 (7·8%) cases due to reduced level of consciousness at onset (17), event related confusion (9), dysphasia (8) or cognitive impairment (3), severe coinciding illness (2) or other causes (13).

**Table 1:** Baseline characteristics

	Pre-FAST (*n* = 416)	Post-FAST (*n* = 252)	*P*-value
Age (mean ± SD)	77·4 (±12·6)	76·6 (±14·7)	0·45
Female sex	238 (57·2)	144 (57·1)	0·99
Caucasian ethnicity	344 (96·4)	208 (94·1)	0·21
Level of education			
Basic	218 (72·7)	108 (57·4)	0·001
Further	59 (19·7)	49 (26·1)	
Higher	23 (7·7)	31 (16·5)	
Socioeconomic status (mean ± SD)*	9·8 (±6·5)	9·6 (±6·1)	0·59
Living alone	136 (36·9)	69 (29·5)	0·06
Stroke severity (NIHSS; median, IQR)	9 (5–15)	8 (5–16)	0·66
Prior stroke	56 (13·5)	24 (9·5)	0·13
Hypertension	230 (58·1)	158 (62·9)	0·22
Diabetes	38 (9·6)	45 (17·9)	0·002
Hyperlipidemia	95 (29·0)	89 (44·1)	<0·001
Smoking			
Never	189 (48·2)	96 (46·4)	0·18
Former	142 (36·2)	88 (42·5)	
Current	61 (15·6)	23 (11·1)	

* Index of deprivation. IQR, interquartile range; NIHSS, National Institutes of Health stroke scale; SD, standard deviation.

Sixty (9·0%) patients could not be properly assessed for presence of FAST symptoms at onset, due to sudden collapse with loss of consciousness. Of the remaining 608 major strokes, 563 (92·6%) had one or more FAST symptoms. Upper limb motor symptoms were noted in 480 (79·5%) cases, speech disturbance in 403 (71·0%), and facial weakness in 356 (60·9%). Of all hemispheric events, although speech disturbance was more common amongst left hemispheric stroke (81·9% vs. 62·6%, OR 2·72, 95% CI 1·78–4·15, *P* < 0·0001), the percentage of FAST-positive cases amongst left vs. right hemispheric strokes did not differ significantly (96·6% vs. 94·2%, OR 1·76, 0·75–4·17, *P* = 0·19).

First medical attention after stroke onset was sought more quickly after April 1, 2009 than before (median [IQR] minutes: before – 53 [15–265] vs. 31 [7–120] after, *P* = 0·005). Attention was more often sought directly via emergency medical services (Table 2, before – 57·2% vs. 74·8% after, OR 2·18 [1·54–3·09], *P* < 0·0001). The decrease in use of non-emergency services (chiefly a general practitioner) declined steeply in 2009 and this change was maintained thereafter (Fig. 2A). This was associated with initiation of the campaign, also after correcting for trend, age, sex, stroke severity, educational level, social class, cohabiting, ethnicity, and prior stroke or TIA (aOR 2·22 [1·05–4·67], *P* = 0·036; Table 3).

**Table 2:** First healthcare provider contacted before and after April 1, 2009

	Pre-FAST (%)	Post-FAST (%)	OR [95% CI]	*P*-value
Non-emergency	172 (42·8)	62 (25·2)	0·45 [0·32–0·65]	<0·0001
GP	168 (41·8)	55 (22·4)	0·40 [0·28–0·58]	<0·0001
NHS-direct	1 (0·2)	2 (0·8)	3·28 [0·30–36·41]	0·31
Other*	3 (0·8)	5 (2·0)	2·76 [0·65–11·64]	0·15
Emergency	229 (57·2)	183 (74·8)	2·20 [1·55–3·13]	<0·0001
A&E	7 (1·7)	9 (3·7)	2·14 [0·79–5·83]	0·13
EMS	222 (55·5)	174 (71·0)	1·97 [1·40–2·76]	<0·0001

* Includes private doctor, consultant review, medical staff whilst on holidays (airport staff, ship doctor, hotel doctor). A&E, Accident & Emergency department; CI, confidence interval; EMS, emergency medical services; GP, general practitioner; NHS-direct, National Health Service helpline; OR, odds ratio.

**Table 3:** Segmented time-series regression analysis for use of emergency medical services and hospital arrival <3 h

	Use of emergency medical services		Hospital arrival within 3 h	
	OR [95% CI]	*P*-value	OR [95% CI]	*P*-value
Constant	2·28	0·19	2·05	0·52
Baseline trend	1·01 [1·00–1·01]	0·16	1·00 [0·99–1·01]	0·33
Change at intervention	2·22 [1·05–4·67]	0·036	3·11 [1·53–6·29]	0·002
Trend after intervention	0·99 [0·97–1·03]	0·34	1·00 [0·98–1·02]	0·99
Age	0·98 [0·97–1·00]	0·015	1·00 [0·99–1·01]	0·94
Female sex	0·83 [0·57–1·21]	0·33	0·79 [0·54–1·16]	0·23
Stroke severity (NIHSS)	1·98 [1·66–2·36]	<0·0001	1·78 [1·51–2·10]	<0·0001
Level of education	0·87 [0·62–1·22]	0·40	0·81 [0·60–1·09]	0·17
Socioeconomic status	0·89 [0·77–1·03]	0·10	0·99 [0·87–1·12]	0·86
Living alone	1·75 [1·17–2·63]	0·007	0·61 [0·41–0·91]	0·014
Ethnicity other than Caucasian	1·16 [0·47–2·90]	0·74	0·73 [0·28–1·92]	0·52
Prior stroke or TIA	0·76 [0·50–1·15]	0·19	0·62 [0·40–0·96]	0·03

Risk estimates were grossly similar when fitting the same model in a complete case analysis (please see online supplement). CI, confidence interval; NIHSS, National Institutes of Health Stroke Scale; OR, odds ratio.

**Figure 1:**
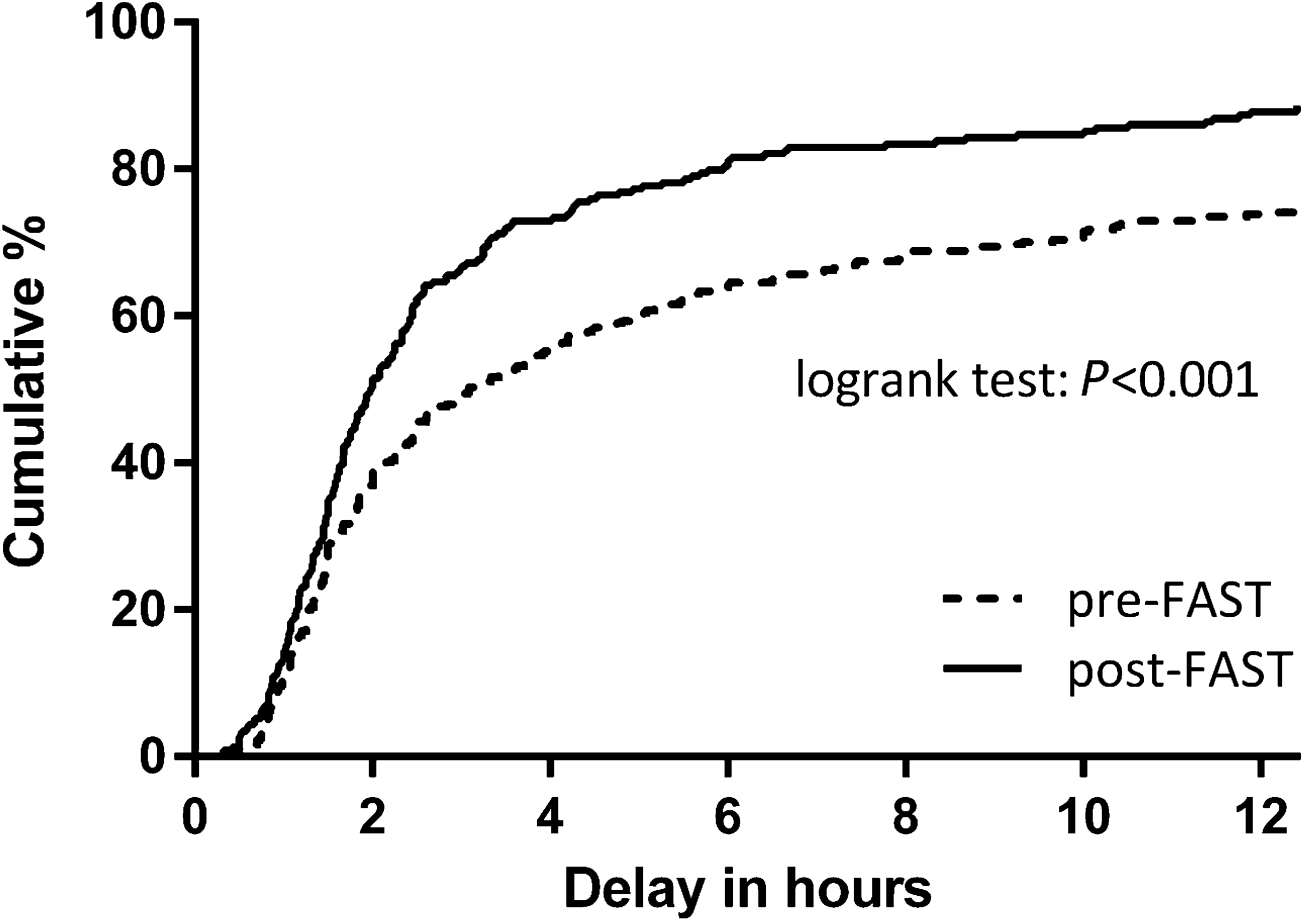
Time to hospital arrival: the percentage of patients that arrived in hospital by time following symptom onset.

**Figure 2:**
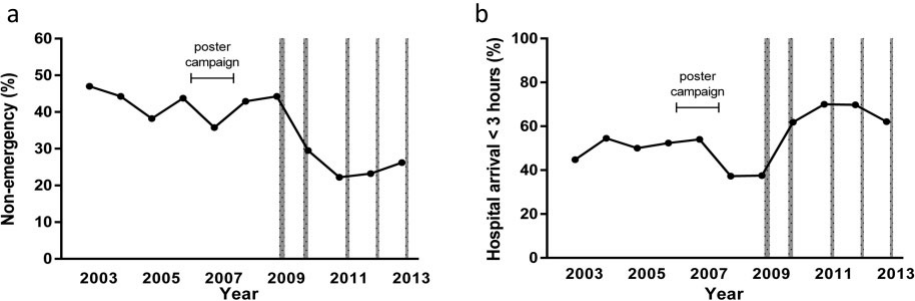
Time-series analysis of patient response to stroke: the frequency (%) of initial non-emergency response throughout the study period (a), and hospital arrival within three-hours (%) throughout the study period (b). The shaded areas reflect episodes of televised FAST campaigning.

The shorter time to seeking medical attention and increased use of emergency medical services after April 1, 2009 resulted in a reduced delay from symptom onset to arrival in hospital (Fig. 1 – logrank: *P* < 0·0001; median [IQR] = 185 [88–885] vs. 119 [78–256] minutes, *P* < 0·0001) and an increase in the proportion of patients reaching hospital within 3 h from 169 (46·9%) to 154 (65·8%) (OR 2·18 [1·55–3·06], *P* < 0·0001). Again, this proportion increased steeply in 2009, this change was maintained thereafter (Fig. 2B) and on time-series analysis significantly associated to initiation of the campaign, also after correcting for trend, age, sex, stroke severity, educational level, social class, cohabiting, ethnicity, and prior stroke or TIA (aOR 3·11 [1·53–6·29], *P* = 0·002; Table 3).

Time to seeking medical attention and hospital arrival were particularly improved in those over 75 years old (within 3 h – OR 2·08 [1·28–3·40], *P* = 0·003, and OR 2·54 [1·66–3·88], *P* < 0·0001, respectively) and those with less education (OR 1·68 [1·02–2·75], *P* = 0·039, and OR 2·26 [1·45–3·52], *P* < 0·0001). The latter group also showed a distinct improvement in use of emergency medical services (OR 2·58 [1·64–4·06], *P* < 0·0001). Results were similar for each quintile of social deprivation within the study population (*P*-value for trend: time to seeking attention – *P* = 0·82; time to hospital arrival – 0·98; use of EMS – *P* = 0·82). Similar results were observed across stroke severity (*P* = 0·38; *P* = 0·12 and *P* = 0·64) and for men and women (*P* = 0·34; *P* = 0·46 and *P* = 0·40). Sensitivity analysis excluding wake-up events were similar to those described above, as were comparisons of right and left hemispheric events, and comparison of the four-years immediately prior to April 1, 2009 vs. the subsequent four-years (please see Supporting information).

Amongst 325 (93·1%) patients with ischaemic stroke after June 1, 2005 for whom data on thrombolytic treatment were available (mean age 78·9 ± 12·3 years, 140 (43·1%) male, median NIHSS 8 [5–14]), there was no difference in the use of thrombolytic treatment after April 1, 2009 (OR 1·37 [0·53–3·58], *P* = 0·52).

Presentation directly to emergency medical services was associated with shortest time to hospital arrival (median 1·6 vs. 10·6 h, please see Fig. S1 in the Supporting information). For patients presenting to emergency medical services, time to seeking medical attention was slightly reduced after April 1, 2009, but there was no change in paramedic response time or ambulance transport time (please see Fig. S1 in the Supporting information). Since April 1, 2009, the mean proportion of pre-hospital delay attributable to paramedic response time and transport to hospital for these patients is 69%.

In 278 (41·6%) patients without severe speech impairment, cognitive impairment or reduced consciousness who provided their initial perception of symptoms {mean age 73·9 ± 14·6 years, 137 (49·3%) male, median NIHSS 6 [4–10]}, 124 (44·6%) correctly attributed these to stroke. Correct patient perception was similar before vs. after April 1, 2009 (46·2% vs. 42·7%; OR 0·87 [0·52–1·44], *P* = 0·58). Overall, correct patient perception was not significantly associated with time to seeking medical attention (median [IQR] minutes: correct – 60 [15–181] vs. incorrect – 60 [15–572], *P* = 0·19) or with the proportion contacting emergency medical services (correct – 52·5% vs. incorrect – 52·4%, OR 1·01 [0·62–1·63], *P* = 0·99). However, in the vast majority of cases (553/617; 89·6%) first medical attention was sought by somebody other than the patient. This proportion was similar before vs. after April 1, 2009 (89·9% vs. 89·2%, OR 0·93 [0·55–1·58], *P* = 0·78). Data on the initial diagnostic impression of these relatives/bystanders were not collected.

## Discussion

Our population-based study shows marked improvement in early presentation after major stroke following the televised FAST campaign in the UK. This appears to be partly attributable to a decrease in time to first seeking medical attention, but most importantly to a shift towards directly contacting emergency medical services. The emphasis on the need for presentation to emergency medical services was a key message of the national UK FAST campaign. Based on data from our study, we estimate that the reduction in delay to hospital arrival would translate into an extra 8000 patients with major stroke in the UK reaching hospital within 3 h.

Increase in use of emergency medical services has repeatedly been cited as one of the most important factors in facilitating rapid medical assessment following stroke 3. In our study, the rate of non-emergency calls following major stroke nearly halved since the FAST campaign. Reports of routinely collected emergency department data and ambulance dispatches from Australia and Ireland identified a short-term increase of emergency presentation for stroke after FAST campaigns 13,14, and routinely collected data in the UK suggest a similar initial trend 20,21. Our study has demonstrated this at the population level and has shown that the apparent effect has been sustained. However, there is still substantial room for improvement, with a recent US study showing no increase in the use of emergency medical services from 2003 till 2010, and around 40% of patients not turning to emergency medical services 16.

Interestingly, patients' recognition of stroke symptoms was unchanged by the FAST campaign in our study and correct recognition was not associated with more urgent behavior. This may in part be explained by cognitive, communicative and motor impairment interfering with any intended response to symptoms. However, since 90% of major stroke patients rely on relatives, acquaintances or other bystanders to call for medical aid, stroke awareness in patients themselves may be of less relevance, highlighting the importance of targeting the general population with educational campaigns about major stroke, rather than high-risk individuals only. The lack of association between patients' perception and behavior for stroke was also found in previous stroke cohorts 22,23. One Korean study amongst 500 stroke patients did find correct perception to be the most important predictor of early hospital arrival 24, but their population consisted mainly of minor stroke patients (median NIHSS 3) who may behave differently 25. Surveys amongst the general population in the UK showed an increase in ability to name stroke warning signs following the FAST campaign 6,26,27, suggesting that improved initial diagnostic impressions in bystanders might have contributed to the positive effects observed in our study, although a small earlier UK study found only a non-significant improvement in time to arrival in hospital 28.

Our study showed that patients initially contacting their general practitioner generally sought medical attention only after the time window for thrombolytic treatment had passed. We also showed that two thirds of pre-hospital delay in patients who did seek emergency medical attention now consists of paramedic assessment and ambulance transport time, highlighting the potential of pre-hospital treatment initiatives 29. The feasibility of future extensive mass-media campaigning will in part depend on its cost-effectiveness. The investment in the televised UK FAST campaign was around £10 million 30. A preliminary assessment of cost-effectiveness concluded every pound sterling spent achieved a payback of £3·20 31. However, these numbers have been disputed based on presumed flaws in the used model, assuming unrealistically high thrombolysis rates 32.

Although we believe our findings are reliable, there are some limitations. First, we are unable to be absolutely certain that the improvements that we observed were due to the FAST campaign. Stroke care in the UK improved more generally during the study period 30, although there were no other public education campaigns. Moreover, the timeframe of the changes we observed fitted well with the timing of the FAST campaign, particularly the change in delays attributable to patients, whereas there were no significant changes in delays attributable to the emergency healthcare system. Second, although we interviewed participants as soon as possible after their stroke, we cannot completely rule out recall bias. Also, we did not record the diagnostic perception of bystanders or relatives, nor did we directly question individual exposure and awareness of the campaign. Third, we were able to adjust for many, but not all factors that have previously been associated with pre-hospital delay 33. Fourth, we did not assess behavior after TIA and minor stroke. Finally, in view of the small fraction of non-Caucasian people in our study (4·5%), the results may not be fully applicable to ethnic minorities, who have previously been reported to delay to presentation longer 28,34.

In conclusion, a large-scale televised national public education campaign about stroke in the UK appears to have led to a sustained improvement in patients' and bystanders' response after major stroke. These findings support continuation of the campaign in the UK and may guide similar public education initiatives in other countries.

## Supporting Information

**Figure S1.** Share of patient delay in median pre-hospital delay in hours by mode of presentation following the FAST campaign (A) and share of patient delay in median pre-hospital delay in hours (B, left) and as a percentage of total pre-hospital delay for emergency presentation before and after the FAST campaign (B, right). **Table S1.** Complete case segmented time-series regression analysis. **Table S2.** Sensitivity analyses assessing wake-up events. **Table S3.** Sensitivity analyses assessing the affected hemisphere. **Table S4.** Sensitivity analyses comparing only four-years directly prior to 2009 vs. four-years after.
