# Supplementary material for: Sustained impact of UK FAST-test public education on response to stroke: a population-based time-series study
Source: Int J Stroke. 2015 Apr 8;10(7):1108–14. doi: 10.1111/ijs.12484 (PMC4672715; doi:10.1111/ijs.12484)
Supplement: Supplementary file 1 — Figure S1. Share of patient delay in median pre-hospital delay in hours by mode of presentation following the FAST campaign (A) and share of patient delay in median pre-hospital delay in hours (B, left) and as a percentage of total pre-hospital delay for emergency presentation before and after the FAST campaign (B, right). Table S1. Complete case segmented time-series regression analysis. Table S2. Sensitivity analyses assessing wake-up events. Table S3. Sensitivity analyses assessing the affected hemisphere. Table S4. Sensitivity analyses comparing only four-years directly prior to 2009 vs. four-years after. [file ijs0010-1108-sd1.docx]

**Online supplement**

**Complete case segmented time-series regression analysis.**

|  | **Use of emergency medical services** | | **Hospital arrival within 3 hours** | |
| --- | --- | --- | --- | --- |
|  | OR [95% CI] | P-value | OR [95% CI] | P-value |
| Constant | 1.88 | 0.51 | 1.44 | 0.72 |
| Baseline trend | 1.01 [1.00-1.02] | 0.14 | 1.00 [0.99-1.01] | 0.65 |
| Change at intervention | 2.38 [0.92-6.13] | 0.073 | 3.14 [1.24-7.94] | 0.015 |
| Trend after intervention | 0.99 [0.96-1.02] | 0.38 | 1.00 [0.97-1.02] | 0.81 |
| Age | 0.99 [0.97-1.00] | 0.11 | 1.01 [0.99-1.02] | 0.53 |
| Female sex | 0.95 [0.60-1.51] | 0.84 | 0.79 [0.49-1.26] | 0.31 |
| Stroke severity (NIHSS) | 1.88 [1.48-2.39] | <0.0001 | 1.80 [1.43-2.27] | <0.0001 |
| Level of education | 0.75 [0.53-1.06] | 0.10 | 0.75 [0.53-1.06] | 0.10 |
| Socioeconomic status | 0.96 [0.93-0.99] | 0.044 | 0.99 [0.95-1.03] | 0.50 |
| Living alone | 1.70 [1.03-2.79] | 0.039 | 0.53 [0.32-0.87] | 0.012 |
| Ethnicity other than Caucasian | 0.74 [0.41-3.47] | 0.74 | 0.62 [0.18-2.17] | 0.45 |
| Prior stroke or TIA | 1.02 [0.61-1.71] | 0.94 | 0.72 [0.42-1.23] | 0.23 |

OR = odds ratio; CI = confidence interval; NIHSS = National Institutes of Health Stroke Scale

Following the complete case analysis, missing data on socio-economic status (9.0%), education (33.4%), and living alone (6.7%) were imputed for the main analysis (Table 3 in the main manuscript). We used fivefold imputation based on the above covariates and outcomes. Variables were distributed similarly before and after imputation, including level of education (basic/further/higher before vs. after: 69.2%/19.3%/11.5% vs. 68.3%/19.7%/12.1%). The segmented time-series regression analysis on the imputed dataset yielded risk estimates similar to the complete case analysis, consequently strengthening the plausibility of missing at random assumption for the missing values.

**Figure A1** Share of patient delay in median pre-hospital delay in hours by mode of presentation following the FAST campaign (A) and share of patient delay in median pre-hospital delay in hours (B, left) and as a percentage of total pre-hospital delay for emergency presentation before and after the FAST campaign (B, right).

**
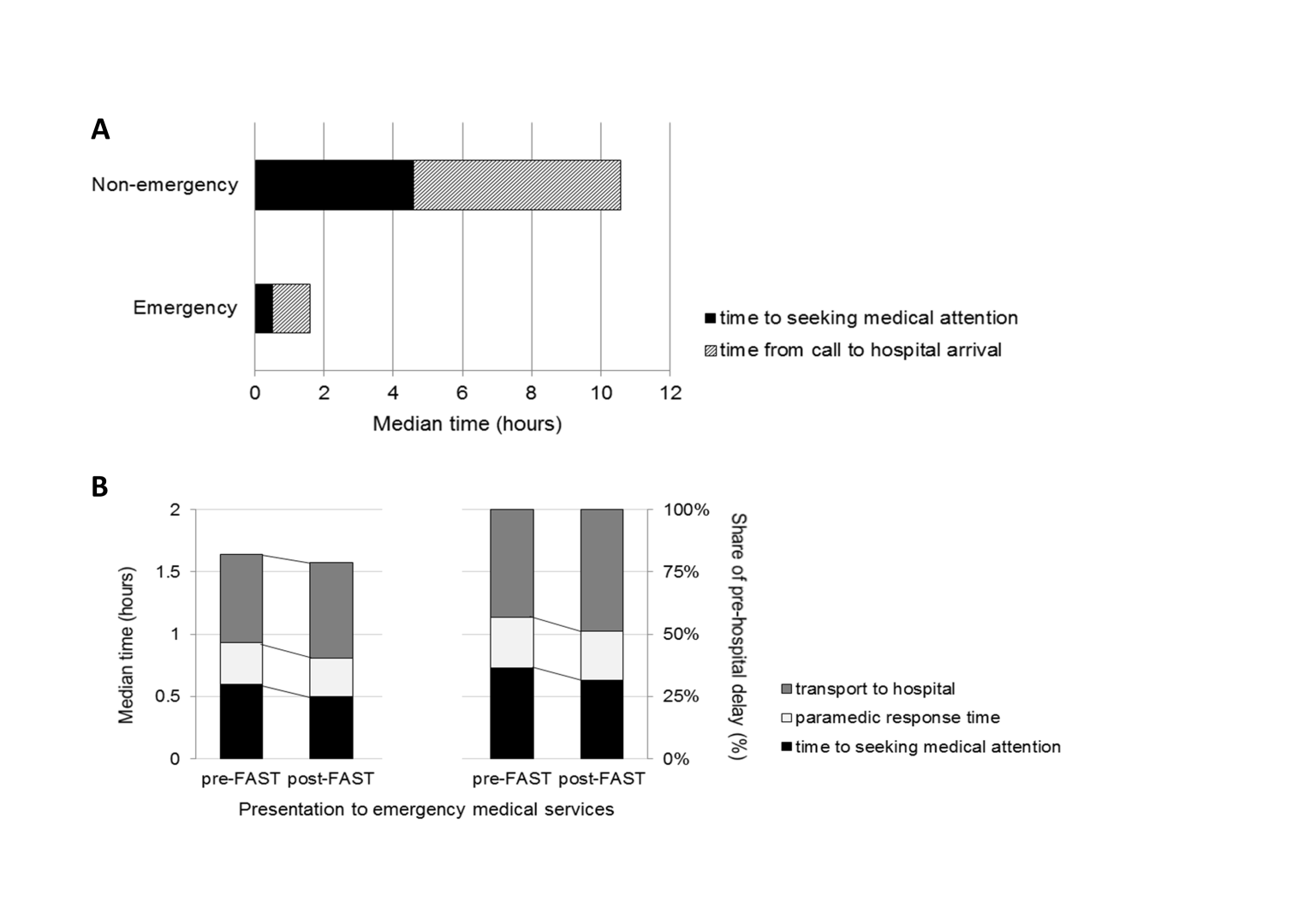
**

**Sensitivity analyses assessing wake-up events.**

|  | Pre-FAST (%) | Post-FAST (%) | OR [95% CI] | P-value |
| --- | --- | --- | --- | --- |
| **Sleep onset** |  |  |  |  |
| Mode of presentation |  |  |  |  |
| Non-emergency | 47 (50.0) | 16 (30.2) | 0.43 [0.21-0.88] | 0.020 |
| Emergency | 47 (50.0) | 37 (69.8) | 2.31 [1.13-4.71] | 0.020 |
|  |  |  |  |  |
| Delay |  |  |  |  |
| Seeking medical attention within 3 hours | 55 (58.5) | 39 (72.2) | 1.85 [0.89-3.80] | 0.09 |
| Time to seeking medical attention (median, IQR) | 2.00 [0.58-6.50] | 1.55 [0.25-4.13] | n/a | 0.14 |
| Hospital arrival within 3 hours | 32 (34.2) | 30 (56.6) | 2.49 [1.25-4.96] | 0.009 |
| Time to hospital arrival (median, IQR) | 4.23 [2.00-29.75] | 2.67 [1.58-8.67] | n/a | 0.063 |
|  |  |  |  |  |
| **Awake onset** |  |  |  |  |
| Mode of presentation |  |  |  |  |
| Non-emergency | 88 (40.2) | 30 (21.1) | 0.40 [0.25-0.65] | <0.0001 |
| Emergency | 131 (59.8) | 112 (78.9) | 2.51 [1.54-4.07] | <0.0001 |
|  |  |  |  |  |
| Delay |  |  |  |  |
| Seeking medical attention within 3 hours | 161 (67.3) | 117 (82.4) | 1.45 [0.85-2.48] | 0.17 |
| Time to seeking medical attention (median, IQR) | 0.50 [0.17-2.25] | 0.42 [0.15-1.50] | n/a | 0.30 |
| Hospital arrival within 3 hours | 110 (53.7) | 97 (69.3) | 1.95 [1.24-3.06] | 0.004 |
| Time to hospital arrival (median, IQR) | 2.47 [1.25-15.50] | 1.78 [1.25-3.40] | n/a | 0.019 |

**Sensitivity analyses assessing the affected hemisphere.**

|  | Pre-FAST (%) | Post-FAST (%) | OR [95% CI] | P-value |
| --- | --- | --- | --- | --- |
| **Left hemispheric events** |  |  |  |  |
| Mode of presentation |  |  |  |  |
| Non-emergency | 81 (44.3) | 25 (21.9) | 0.35 [0.21-0.60] | <0.0001 |
| Emergency | 102 (55.7) | 89 (78.1) | 2.83 [1.66-4.81] | <0.0001 |
|  |  |  |  |  |
| Delay |  |  |  |  |
| Seeking medical attention within 3 hours | 124 (72.9) | 88 (78.6) | 1.36 [0.77-2.39] | 0.28 |
| Time to seeking medical attention (median, IQR) | 0.58 [0.25-3.17] | 0.45 [0.12-2.00] | n/a | 0.064 |
| Hospital arrival within 3 hours | 89 (51.7) | 74 (68.5) | 2.03 [1.23-3.36] | 0.006 |
| Time to hospital arrival (median, IQR) | 2.55 [1.43-10.00] | 1.81 [1.25-4.22] | n/a | 0.007 |
|  |  |  |  |  |
| **Right hemispheric events** |  |  |  |  |
| Mode of presentation |  |  |  |  |
| Non-emergency | 52 (38.8) | 24 (29.6) | 0.66 [0.37-1.19] | 0.17 |
| Emergency | 82 (61.2) | 57 (70.4) | 1.51 [0.84-2.72] | 0.17 |
|  |  |  |  |  |
| Delay |  |  |  |  |
| Seeking medical attention within 3 hours | 86 (67.2) | 65 (81.2) | 2.11 [1.08-4.14] | 0.027 |
| Time to seeking medical attention (median, IQR) | 1.00 [0.25-6.43] | 0.79 [0.17-2.25] | n/a | 0.10 |
| Hospital arrival within 3 hours | 50 (42.7) | 47 (60.3) | 2.03 [1.13-3.64] | 0.017 |
| Time to hospital arrival (median, IQR) | 3.74 [1.50-20.42] | 2.25 [1.43-4.27] | n/a | 0.021 |

**Sensitivity analyses comparing only 4 years directly prior to 2009 versus 4 years after.**

|  | Pre-FAST (%) | Post-FAST (%) | OR [95% CI] | P-value |
| --- | --- | --- | --- | --- |
| **Data from 2005-2013** |  |  |  |  |
| Mode of presentation |  |  |  |  |
| Non-emergency | 91 (41.7 | 62 (25.3) | 0.47 [0.32-0.70] | <0.0001 |
| Emergency | 127 (58.3) | 183 (74.7) | 2.12 [1.43-3.14] | <0.0001 |
|  |  |  |  |  |
| Delay |  |  |  |  |
| Seeking medical attention within 3 hours | 143 (69.4) | 192 (79.3) | 1.69 [1.10-2.60] | 0.016 |
| Time to seeking medical attention (median, IQR) | 1.00 [0.25-4.75] | 0.52 [0.12-2.00] | n/a | 0.004 |
| Hospital arrival within 3 hours | 89 (44.7) | 154 (65.8) | 2.38 [1.61-3.51] | <0.0001 |
| Time to hospital arrival (median, IQR) | 3.33 [1.50-11.40] | 1.98 [1.30-4.27] | n/a | <0.0001 |
|  |  |  |  |  |
| Perception |  |  |  |  |
| Correct | 40 (40.8) | 38 (42.7) | 1.08 [0.60-1.93] | 0.80 |
| Incorrect | 58 (59.2) | 51 (57.3) |  |  |
